# Interacting Symbionts and Immunity in the Amphibian Skin Mucosome Predict Disease Risk and Probiotic Effectiveness

**DOI:** 10.1371/journal.pone.0096375

**Published:** 2014-04-30

**Authors:** Douglas C. Woodhams, Hannelore Brandt, Simone Baumgartner, Jos Kielgast, Eliane Küpfer, Ursina Tobler, Leyla R. Davis, Benedikt R. Schmidt, Christian Bel, Sandro Hodel, Rob Knight, Valerie McKenzie

**Affiliations:** 1 Institute of Evolutionary Biology and Environmental Studies, University of Zurich, Zurich, Switzerland; 2 Department of Ecology and Evolutionary Biology, University of Colorado, Boulder, Colorado, United States of America; 3 Section for Freshwater Biology, Department of Biology, University of Copenhagen, Copenhagen, Denmark; 4 Department of Evolutionary Biology, Technical University of Braunschweig, Braunschweig, Germany; 5 KARCH, Neuchâtel, Switzerland; 6 Howard Hughes Medical Institute and Department of Chemistry and Biochemistry, BioFrontiers Institute, University of Colorado, Boulder, Colorado, United States of America; University of Sao Paulo, Brazil

## Abstract

Pathogenesis is strongly dependent on microbial context, but development of probiotic therapies has neglected the impact of ecological interactions. Dynamics among microbial communities, host immune responses, and environmental conditions may alter the effect of probiotics in human and veterinary medicine, agriculture and aquaculture, and the proposed treatment of emerging wildlife and zoonotic diseases such as those occurring on amphibians or vectored by mosquitoes. Here we use a holistic measure of amphibian mucosal defenses to test the effects of probiotic treatments and to assess disease risk under different ecological contexts. We developed a non-invasive assay for antifungal function of the skin mucosal ecosystem (mucosome function) integrating host immune factors and the microbial community as an alternative to pathogen exposure experiments. From approximately 8500 amphibians sampled across Europe, we compared field infection prevalence with mucosome function against the emerging fungal pathogen *Batrachochytrium dendrobatidis*. Four species were tested with laboratory exposure experiments, and a highly susceptible species, *Alytes obstetricans*, was treated with a variety of temperature and microbial conditions to test the effects of probiotic therapies and environmental conditions on mucosome function. We found that antifungal function of the amphibian skin mucosome predicts the prevalence of infection with the fungal pathogen in natural populations, and is linked to survival in laboratory exposure experiments. When altered by probiotic therapy, the mucosome increased antifungal capacity, while previous exposure to the pathogen was suppressive. In culture, antifungal properties of probiotics depended strongly on immunological and environmental context including temperature, competition, and pathogen presence. Functional changes in microbiota with shifts in temperature provide an alternative mechanistic explanation for patterns of disease susceptibility related to climate beyond direct impact on host or pathogen. This nonlethal management tool can be used to optimize and quickly assess the relative benefits of probiotic therapies under different climatic, microbial, or host conditions.

## Introduction

Probiotic therapies often aim to extend or shape the immune function of hosts by altering the symbiotic microbial community. Probiotics are used in human and veterinary medicine, agriculture and aquaculture, and have been proposed for treatment of emerging wildlife diseases such as those occurring on corals and amphibians [1], [2]. Microbiota can mediate pathogenesis through a range of mechanisms [3], [4], and disease ecology studies demonstrate that parasitic and non-parasitic microbes interact with each other and with the host immune system such that pathogenicity is often influenced by environmental conditions [5]–[8]. Thus, the environment affects the risk of disease to individuals, populations, and species, and assessing disease risk under changing conditions is vital to conservation and infectious disease mitigation and can direct the allocation of resources for most effect [9]–[12].

The microbiota inhabiting skin and mucosal surfaces has a profound impact on host health and immunity [7], [13], [14], and may be predictive of risk for some diseases [15]–[17]. Amphibian skin is a model system for diseases affecting vertebrate mucosa. The mucosome, or micro-ecosystem of the mucus, as defined here contains interdependent host factors (mucosal antibodies, antimicrobial peptides, lysozyme, alkaloids) and microbial-community factors (microbiota, antibiotic metabolites). The mucosome has various functions potentially including communication, and predator and pathogen defense. Here, we develop a non-lethal assay and holistic measure referred to as “mucosome function” to describe the effect of amphibian skin mucus on pathogen viability. We examine how environmental and immunological contexts may impact the outcome of host-microbe symbioses, and how mucosome function captures the *in vivo* complexity of the micro-ecosystem and can thus accurately predict susceptibility to infection. We focus on probiotic bacteria and fungi applied to the skin mucosome as biocontrol agents against the emerging amphibian disease chytridiomycosis.

Chytridiomycosis is a major cause of global amphibian population declines and species extinctions [18], [19]. The disease is caused by the chytrid fungus *Batrachochytrium dendrobatidis*, or *Bd*, and is strongly influenced by climatic conditions [20]. Climate-linked changes to the entire microbiota, not just *Bd*, may influence disease susceptibility [5]. Current efforts to mitigate chytridiomycosis in wildlife populations have turned to bioaugmentation, or the use of probiotic therapies [1], [21]. The successful prophylactic use of *Janthinobacterium lividum* was demonstrated against chytridiomycosis in mountain yellow-legged frogs, *Rana muscosa* [22]. However, when tested on the endangered Panamanian golden frog, *Atelopus zeteki*, the probiotic survived briefly on the skin, but did not protect the amphibians from disease [23]. Similarly, the probiotic *Pedobacter cryoconitis* temporarily reduced infection loads of heavily infected *R. muscosa* [24]. Each target host may thus require probiotic therapy tailored to that species, population, or life-history stage. Screening the various bacteria associated with hosts or their environment to identify effective probiotics is challenging [25], [26]. Thus, probiotic therapies for amphibians must be optimized, and an understanding of which candidate bacteria can establish and persist on the host in its natural environmental context is urgently needed.

To date, all attempts to apply probiotic therapy against chytridiomycosis have used simple selection criteria for choosing candidate probiotics. Selection of the most efficient probiotic is challenging because there are hundreds of culturable phylotypes to choose from, either from environmental sources, or more typically, from tolerant host populations that can persist with nonlethal *Bd* infections [1]. However, simple co-culture assays to determine antifungal capacity have been insufficient to ensure probiotic effectiveness [23], [24]. Co-factors including interactions of the probiotic with the microbial community already present on the amphibian skin, as well as interactions with host immune defenses, and effects of environmental conditions, may complicate the outcome of biotherapy. Here, we experimentally test the impact of immunological and environmental context on potential probiotic bacteria both *in vitro* and *in vivo*. The tested conditions are illustrative rather than comprehensive for potential environmental conditions, community and immunological interactions. Because it is impractical to test all potential interactions before testing probiotics on amphibians for a disease resistance effect, we suggest a protocol for selecting probiotics with the highest potential benefit, and to test whether the probiotics will likely be effective in the range of foreseeable conditions on the host. Our non-lethal susceptibility assay of mucosome function can help assess disease risk and treatment effects in rare amphibians including relict populations or captive populations of endangered species intended for reintroduction.

Typical approaches to compare species susceptibility and to assess disease risk include pathogen exposure experiments [27], or field surveys to compare infection prevalence and monitor disease and population trajectories [28], or modeling environmental and biogeographic risk factors [10], [29]. Deficits of conventional pathogen exposure experiments include lack of environmental context when amphibians are exposed under clean laboratory conditions. Biodiversity including microbiota and macrobiota can influence disease outcome [30], and bacterial community diversity is reduced through time in captivity without natural sources such as soil for re-inoculating the skin [31]. The exposure history, population genetics, and life-history stage of the amphibians used in the experiment, as well as the strain and dose of the pathogen can all affect experimental outcomes, and many threatened species are not suitable for such experiments. In addition, growth of *Bd* is often inhibited by skin microbiota of amphibians [32], [33]. However, little is known about how protective microbiota differs among host populations or regions, or how mucosome function is altered by enrichment with potential probiotics.

Our aims in this study were (1) to develop a holistic, simple, non-invasive, and non-lethal method to measure mucosome function against *Bd*. Using this tool, we aimed (2) to test whether mucosome function can predict *Bd* infection prevalence of amphibians in the field and survival in *Bd* exposure experiments. While we show that probiotics are influenced by a variety of factors including competition, temperature, and innate immunity when tested *in vitro*, we aimed (3) to use mucosome function as an ecologically-integrated predictor of probiotic therapy effect so that future research can test probiotic strategies for conservation and not lose hope in the potential of probiotic therapy in the face of immunological and ecological complexity. We provide a detailed protocol for measuring mucosome function in File S1.

## Materials and Methods

### Ethics statement

Permits to conduct fieldwork were obtained from the Swiss cantonal conservation authorities, and from Germany - German federal licence (Rheinland-Pfalz) no. 425-104.143.0904 Struktur- und Genehmigungsdirektion Nord, Koblenz. All animal procedures were approved by the Veterinary Authority of Zurich (110/2007 and 227/2007) and the Federal Office for the Environment. Fieldwork conformed to standard decontamination practices to avoid transport of pathogens between sites. All animals in experiments were monitored daily for animal welfare and to ameliorate suffering. During experiments, any individual demonstrating clinical signs of disease including lethargy, abnormal skin shedding, and loss of righting reflex were humanely euthanized. At the end of the experiment, all animals were humanely euthanized by overdose of tricaine methanesulfonate.

### Survey of *Bd* infection prevalence

To compare *Bd* infection prevalence among species and life-history stages, we combine previously unpublished results from field studies in Switzerland with *Bd* surveys from amphibians across Europe collated by Bd-Maps (www.bd-maps.net, accessed September 1, 2013). In addition to data from 5939 sampled amphibians available from Bd-maps, skin swabs were collected from 2591 amphibians from 12 species and from 66 *Bd*-positive populations from the northern parts of Switzerland and tested for *Bd* between 2007 and 2009 (Table 1). Amphibians were caught by dip-netting and swabbed with a sterile cotton swab (Copan Italia S.p.A., Brescia, Italy). Field material was cleaned and disinfected before moving between different sites to avoid contamination and spread of *Bd* and other pathogens. Extraction and analysis for *Bd*-DNA were done following the qPCR protocol by Boyle *et al.* [34] using *Bd*-specific primers and standards to quantify the amount of DNA. We ran each sample twice and the PCR was repeated if the two wells returned dissimilar results. Reactions below 1 genomic equivalent were scored *Bd*-negative to avoid false positives. Mean infection prevalence with 95% binomial confidence interval was calculated for each species and life stage sampled, and calculated for both Europe and Switzerland.

**Table 1:** Amphibians from Switzerland sampled for skin peptide effectiveness and mucosome function against *Bd*, and *Bd* infection prevalence at different life-history stages.

Species	Life-history stage#	Peptide effectiveness* (N)	SE	Mean mucosome function against Swiss *Bd* (N)	SE	Switzerland: Percent infected (N)	95% binomial confidence interval	Europe: Percent infected (N)	95% binomial confidence interval
*Alytes obstetricans*	Adult/Subadult	15.92 (8)	6.21	0.012 (10)	0.000	4.9 (41)	0.6–16.5	29.7 (209)$	23.5–36.4
*Alytes obstetricans*	Metamorph	37.75 (5)	12.15						
*Alytes obstetricans*	Larvae	48.76 (5)	24.23	2.963 (10)	0.681	45.4 (2111)	43.3–47.6	38.0 (3008)	36.3–39.8
*Bombina variegata*	Adult/Subadult			1.075 (4)	0.081	20.0 (150)	13.9–27.3	21.1 (227)	16.0–27.0
*Bufo bufo*	Adult	16.34 (15)	3.37753	0.117 (9)	0.082	0.0 (22)	0.0–15.4	0.9 (3606)	0.6–1.2
*Bufo bufo*	Larvae			1.284 (5)	0.404			6.7 (45)	1.4–18.3
*Hyla arborea*	Adult	11.42 (7)	2.15210			3.8 (26)	0.1–19.6	12.5 (32)	3.5–29.0
*Ichthyosaura alpestris*	Adult	0.94 (7)	.52546	1.361 (20)	0.062	24.8 (629)	21.5–28.4	21.5 (775)	18.7–24.6
*Lissotriton vulgaris*	Adult	1.85 (4)	1.02506			27.3 (22)	10.7–50.2	17.0 (47)	7.7–30.8
*Pelophylax lessonae/esculentus*	Adult	27.27 (10)	3.18			22.4 (170)	16.3–29.4	15.6 (275)	11.6–20.5
*Pelophylax lessonae/esculentus*	Metamorph	5.34 (5)	1.88685	0.545 (10)	0.042	13.0 (69)	6.1–23.3	13.2 (76)	6.5–22.9
*Rana temporaria*	Adult/Subadult	1.97 (13)	.62111	0.251 (10)	0.128	0.0 (10)	0.0–30.9	3.1 (129)	0.9–7.8
*Rana temporaria*	Larvae			0.220 (5)	0.120	0.0 (20)	0.0–16.8	0.0 (23)	0.0–14.8
*Salamandra salamandra*	Adult	4.92 (9)	1.32654					11.1 (9)	0.3–48.3
*Salamandra salamandra*	Larvae	42.78 (5)	13.35528					23.2 (69)	13.9–34.9

Skin peptide effectiveness is the percent inhibition of *Bd* zoospore growth caused by 50 µg/ml peptide multiplied by the quantity of peptides (mg) per g amphibian according to Woodhams et al. [11]. The mucosome function against *Bd* (Swiss isolate TG 739) is a measure of zoospore viability quantified by the ratio of green:red fluorescence as described above. Infection prevalence is the mean from all amphibians in each group from multiple sites and seasons.

# Larval and post-metamorphic skin peptide samples extracted by different methods.

*Peptide effectiveness  = % inhibition of *Bd* growth at 50 µg/ml * mg peptides/g frog mass.

$ Includes samples from chytridiomycosis outbreak sites in Spain (S. Walker, unpubl.), not included in logistic regression.

### *Bd* infection prevalence predicted by skin defenses

Skin defense peptides and mucosome samples were tested against *Bd* for comparison of anti-*Bd* activity with infection prevalence in natural populations by logistic regression in R. Amphibians sampled for skin peptides and mucosome function (Table 1) were sampled in Switzerland and compared to field infection prevalence from Switzerland and across Europe in separate analyses. Skin peptides were collected upon induction by subcutaneous injection of metamorphosed amphibians with 40 nmole/g body mass norepinephrine (bitartrate salt, Sigma) or immersion of larval amphibians in 100 µM norepinephrine, and tested for *Bd* growth inhibition as previously described [35], [36]. Skin peptide samples from post-metamorphic amphibians only were used in the logistic regression analyses because different methods of peptide induction were used on larval stages. Mucosome samples from multiple life-history stages of the same species were included and matched to life-history stages sampled for *Bd* diagnostics (Table 1). Detailed methods for measuring mucosome function against *Bd* using a fluorescence assay of *Bd* viability adapted from Stockwell *et al.* [37] (Fig. S1 in File S1) and comparisons of mucosome function and skin peptide defenses against *Bd* are presented in Supporting Information (Figs. S4, S5 in File S1).

### Survival predicted by mucosome function

To examine the relationship between mucosome function against *Bd* and susceptibility to infection and subsequent survival we performed experimental exposures to *Bd* on four species. All animals were exposed to zoospores from Swiss lineage *Bd* TG 739 isolated from a moribund *A. obstetricans* in Gamlikon, Switzerland in 2007 [38] and cryopreserved until use. Egg clutches were obtained from *P. esculentus* (n = 8), *B. variegata* (n = 8), *R. temporaria* (n = 45), and *A. obstetricans* (n = 13) in northern Switzerland or southern Germany. *Rana temporaria* were raised in outdoor mesocosms through metamorphosis before experimental exposure of metamorphs to *Bd* (N = 92 exposed, 94 control). Other species were exposed to *Bd* as tadpoles (N = 80 exposed, 40 control per species). All animals were kept in the same laboratory at 19°C during the experiments. We measured the proportion of infected metamorphs by qPCR, and determined relative survival (survival of infected/survival of uninfected controls) at the end of the experiments (50–90 d after metamorphosis). Kaplan Meier curves are presented in the Supporting Information for each species. We examined the relationship between mucosome function against *Bd* and relative survival and proportion infected using logistic regression analyses in R.

### Host ecological context and skin defenses

The *in vivo* effects of ambient temperature and skin microbiota on mucosome function against *Bd* and skin peptide defenses were tested on a focal amphibian species, *A. obstetricans*. In Europe, the common midwife toad, *A. obstetricans*, is a species of conservation concern [39] and is particularly sensitive to *Bd* early in life-history [40]. Host-associated bacteria and fungi were surveyed by culturing from populations of midwife toads near Basel, Switzerland in May, 2009, including samples from 19 adults, 32 larvae, and 9 egg clutches. Although many diverse antifungal bacteria have been described in association with skin of some amphibian hosts [32], [33], we chose eleven bacterial residents isolated from *A. obstetricans* for the environmental context experiments described below based on potency against *Bd* in culture and high prevalence in the populations sampled (L. Davis, unpublished). Two bacterial isolates with high *in vitro* potency against *Bd* and the ability to withstand host skin defense peptides, and one fungal isolate, were chosen for applications on recently metamorphosed *A. obstetricans*.

All metamorphs used in the study were raised in captivity from wild-caught tadpoles that were naturally exposed to the fungus in their pond of origin, near Zunzgen, Switzerland, but negative for *Bd* by qPCR at the time of the experiment. Toadlets were of similar size (mean±SD: 2.1±0.3 g; ANOVA F_5_ = 1.179, *P* = 0.332) and treated at the same time with one exception. Toadlets in the *Bd*-exposure group were exposed to *Bd* approximately 2 months prior to the microbial exposure treatments, and the toadlets were smaller (1.5±0.3 g), and no longer infected at the time of sampling based on qPCR.

We treated recently metamorphosed common midwife toads, *Alytes obstetricans* (N = 70: 10 per treatment group, 7 treatments), by housing them individually at 5°C, 18°C, or 25°C with no microbes added, or at 18°C with exposure to *Bd* zoospores (8.5×10^6^ zoospores of global panzootic lineage isolated from a *Bufo bufo* in the UK [38]), a probiotic fungus *Penicillium expansum*, or a probiotic bacterium *P. fluorescens* or *F. johnsoniae*. Toadlets were bathed individually for one hour in water containing the microbes and after 2 weeks, toadlets from all treatments were sampled on the same day for mucosome function and subsequently skin peptides, sampled as described above.

### Temperature, competition of probiotic strains, and co-culture with *Bd*

To determine the effects of competitive interactions and temperature on probiotic potential, 11 common host-associated isolates were chosen. These included two isolates of *Serratia plymuthica* and one isolate of *Janthinobacterium lividum* from egg clutches of midwife toads, three isolates of *Flavobacterium johnsoniae* and five species of *Pseudomonas* isolated from the skin of adults. Based on 16S rRNA gene sequences, all 11 isolates were considered unique operational taxonomic units (OTUs) at 99%, but clustered into 7 OTUs at 97% similarity as determined by the UCLUST algorithm in QIIME. The 16S rRNA gene sequences of all isolates were deposited in the European Nucleotide Archive (Table S1 in File S1).

In one set of experiments, bacterial isolates were freshly grown at 18°C on RIIA agar media supplemented with 1% tryptone then transferred to experimental conditions. Bacteria and *Bd* (Swiss isolate TG 739) readily grew on the same media. Plate experiments were performed in duplicate. Both isolates of *Serratia plymuthica* were grown separately at 18 and 25°C, or at 18°C on media inoculated with *Bd* and allowed to dry before streaking the bacteria. Two isolates of *F. johnsoniae* were grown separately or combined on media inoculated with *Bd*, and grown at 18°C. When combined, each isolate was streaked across the entire plate. Three *Pseudomonas* isolates were grown either separately, combined, or combined on media inoculated with *Bd*, and grown at 18°C. Control plates of sterile media or *Bd*-only were also tested. All plates were incubated for 3 days, and then rinsed with 2 ml sterile Mili-Q water. Rinse water was then filtered through a 0.22 µm syringe filter.

Bacteria were also grown in liquid RIIA media for 4 d at 14, 19, and 22°C, and metabolites filtered as above. Metabolites from liquid cultures were added to *Bd* zoospores (Global panzootic lineage VMV 813 from a bullfrog, *Lithobates catesbeianus* tadpole) to test for inhibitory effects on pathogen growth. To determine the effect of bacterial filtrate on *Bd* growth, *Bd* zoospores were harvested in 1% tryptone and counted under a hemocytometer. Wells of a 96-well plate were inoculated with 50 µl zoospores at 8×10^6^ zoospores per ml. Then, 50 µl of filtrate (or filtrate diluted 1∶10) from each of the experimental or control plates, or liquid cultures, was added to the wells in replicates of four. In addition, 6 positive control wells contained *Bd* and 50 µl sterile water or RIIA media, and 6 negative control wells contained heat-killed *Bd* and 50 µl sterile water or RIIA media. The change in optical density measured at 490 nm absorbance over 7 days growth at 19°C was recorded using a Victor3 multilabel plate reader (PerkinElmer). Standard statistical testing was carried out in IBM SPSS Statistics 22. Significant *Bd* growth inhibition (or enhancement) caused by bacterial filtrate was determined by t-test, and a repeatable result (Table S2 in File S1). Percent inhibition depended on filtrate dose (see Results) and was not considered comparable among bacterial isolates.

### Effects of host skin peptides and *Bd* metabolites on probiotics in culture

To test for the response of bacterial growth upon culture with either *Bd* filtrate or host skin peptides, bacteria were grown in RIIA liquid media on 96 well plates. Supernatant from a 2-week old culture of *Bd* (type isolate JEL 197) growing in 0.5% tyrptone was filtered through a 0.22 µm syringe filter. An equal volume of *Bd* filtrate or sterile media was added to bacterial cultures. To test effects of peptides, we added an equal volume of sterile water or natural mixtures of partially-purified skin peptides from *A. obstetricans* metamorphs at a final concentration of 100 µg/ml in water. Growth after 48 hr was measured as change in optical density measured at 480 nm. Differences between experimental and control bacterial growth were tested by t-tests using a Bonferroni correction for multiple comparisons.

## Results

### Survey of *Bd* infection prevalence

**Figure 1:**
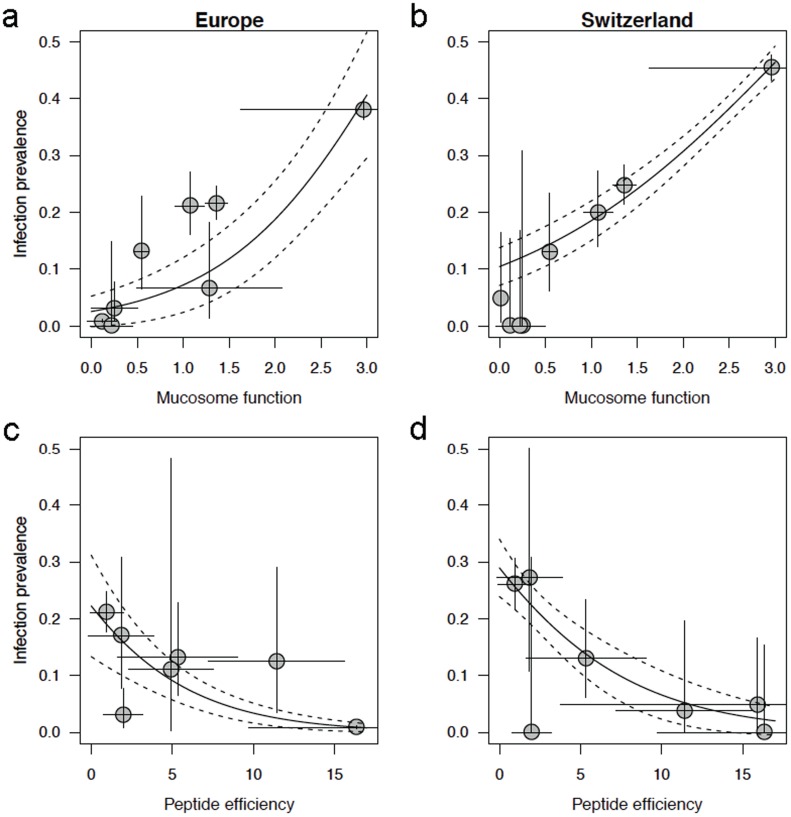
Infection prevalence (mean, 95% binomial CI) of amphibians sampled across Europe and within Switzerland predicted by mucosome function and skin defense peptide activity against *Batrachochytrium dendrobatidis* (*Bd)* zoospores. Mucosome function (mean, SE) indicates *Bd* viability after a 1 hr exposure to amphibian mucus (a,b) and units represent green:red fluorescence. Peptide efficiency (mean, SE) indicates quantity of natural mixtures of skin peptides induced from granular glands multiplied by activity of a standard concentration of peptides against *Bd* zoospore growth. Only post-metamorphic amphibians sampled upon subcutaneous injection with norepinephrine are plotted in (c) and (d). Amphibian skin mucosome function is a better predictor of infection prevalence than induced skin peptide efficiency (logistic regression, see text). Summary data for all species and life-history stages are presented in Table 1.

Surveys of approximately 8500 amphibians (http://www.bd-maps.net/; this study) at different life-history stages for *Bd* infection based on qPCR indicated high prevalence in larval midwife toads, *A. obstetricans* (45% infected in Switzerland) and aquatic adult newts *Ichthyosaura alpestris* (26%), and *Lissotriton vulgaris* (27%). Low infection prevalence (<5%) was detected in populations of adult *A. obstetricans*, *Bufo bufo*, *Rana temporaria*, and *Hyla arborea* (Table 1, Fig. 1).

### *Bd* infection prevalence predicted by skin defenses

We examined two non-lethal measures of susceptibility to infection in pathogen-free Swiss amphibians acclimated to laboratory conditions. These included testing *Bd* growth or viability upon exposure to natural mixtures of partially purified skin defense peptides, and a holistic functional measure of the skin mucosal ecosystem (mucosome function) including ambient skin defenses: peptides, alkaloids, lysozymes, mucosal antibodies, microbiota and microbial metabolites [41]. Both antifungal skin peptides and mucosome function were correlated with infection prevalence in natural populations across Europe (Fig. 1a,c) and within Switzerland (Fig. 1b,d). Prevalence of infection with *Bd* decreased with peptide efficiency (Fig. 1c,d, logistic regressions: Europe, P = 0.0015; Switzerland, P = 0.0079). While induced peptide defenses stored in granular glands were measured here, ambient peptides (not induced by norepinephrine) are a natural component of the mucosome [42], [43]. Mucosome function was tightly correlated to *Bd* prevalence in natural populations of Swiss amphibians (Fig. 1b, P<0.0001) and in amphibians across Europe (Fig. 1a, P = 0.0020). The odds ratios of *Bd* colonization in Swiss amphibians was 1.950 (Europe, 2.969) with each unit change in mucosome function, and 0.839 (Europe, 0.811) with each unit decrease in skin peptide efficiency. Correlations of mucosome function and induced skin peptide efficiency are presented in Figure S4 in File S1 and suggest that both host and microbial factors contribute to mucosome function against *Bd*.

### Survival predicted by mucosome function

**Figure 2:**
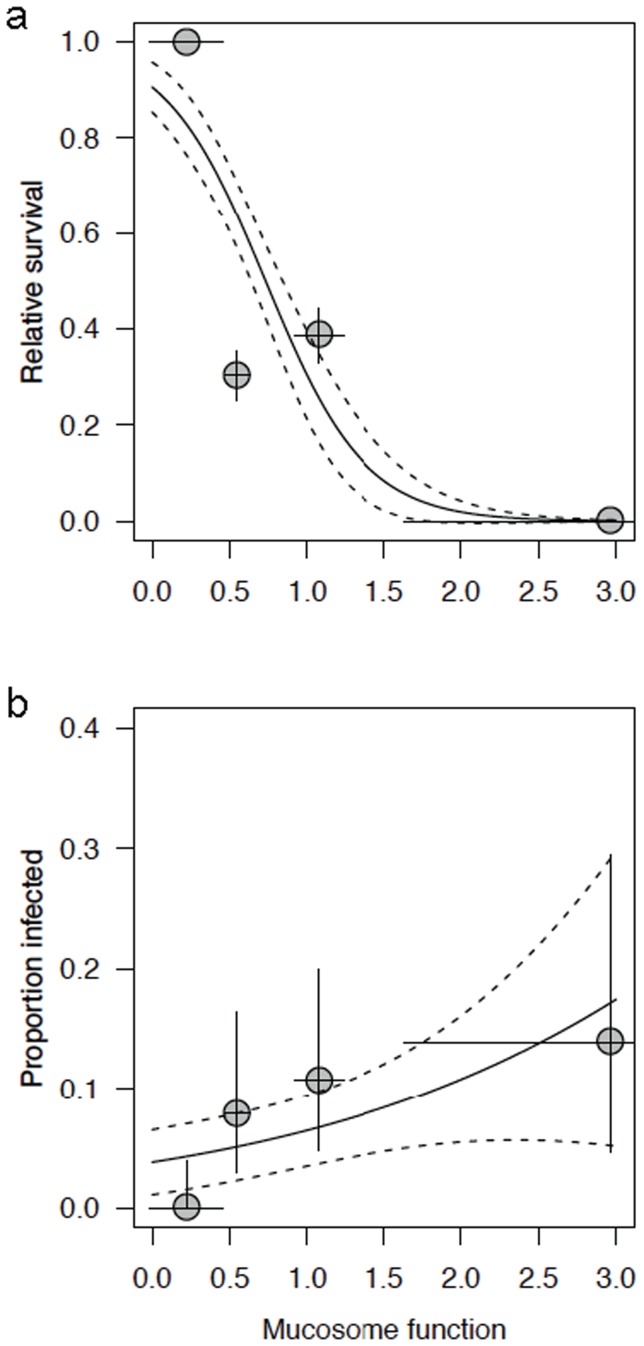
Relative survival (95% binomial CI; a) and Proportion of infected frogs (95% binomial CI; b) predicted by Mucosome function. Post-metamorphosis survival was measured from four Swiss amphibian species after exposure to zoospores of a Swiss *Bd* isolate, TG 739. Survival curves for each species are presented in Supporting Information (Figs. S2, S3 in File S1) and relative survival was calculated as the proportion of infected frogs surviving/proportion of unexposed control frogs surviving. *Alytes obstetricans* showed the highest infection and mortality, and *Rana temporaria* the lowest, with *Bombina variegata* and *Pelophylax esculentus* intermediate. All frogs were raised in captivity from egg clutches and had no history of natural exposure to *Bd*. Mucosome function (mean, SE) indicates *Bd* viability after exposure to amphibian mucus and is a significant predictor of both survival (binomial logistic regressions, P<0.0001) and infection prevalence (P = 0.0106).

Pathogen exposure experiments were conducted on four host species with a Swiss isolate of *Bd*, and relative survival post-metamorphosis of infected tadpoles differed among species (% relative survival, mean±SD days survived): *A. obstetricans* (0%, 24±17.5 d), *Bombina variegata* (39.0%, 32±23.9 d), and *Pelophylax esculentus* (30.4%, 12±12.8 d; Fig. S2 in File S1). Relative survival of recently metamorphosed *Rana temporaria* exposed to *Bd* was 100% (Fig. S3 in File S1), and no colonization by *Bd* was detected by qPCR (n = 92). Success of *Bd* colonization of tadpoles also differed among species (Pearson χ^2^_3_ = 13.102, P = 0.004): *A. obstetricans* (13.9% infected, n = 36), *B. variegata* (10.7%, n = 75), and *P. esculentus* (7.9%, n = 76). Mucosome function predicted survival (logistic regression, P<0.0001; Fig. 2a) and infection with *Bd* in these species (P = 0.0106; Fig. 2b). The odds of infection increased by 1.751 with each unit change in mucosome function, and the odds of survival decreased by 0.0454.

### Host ecological context and skin defenses

**Figure 3:**
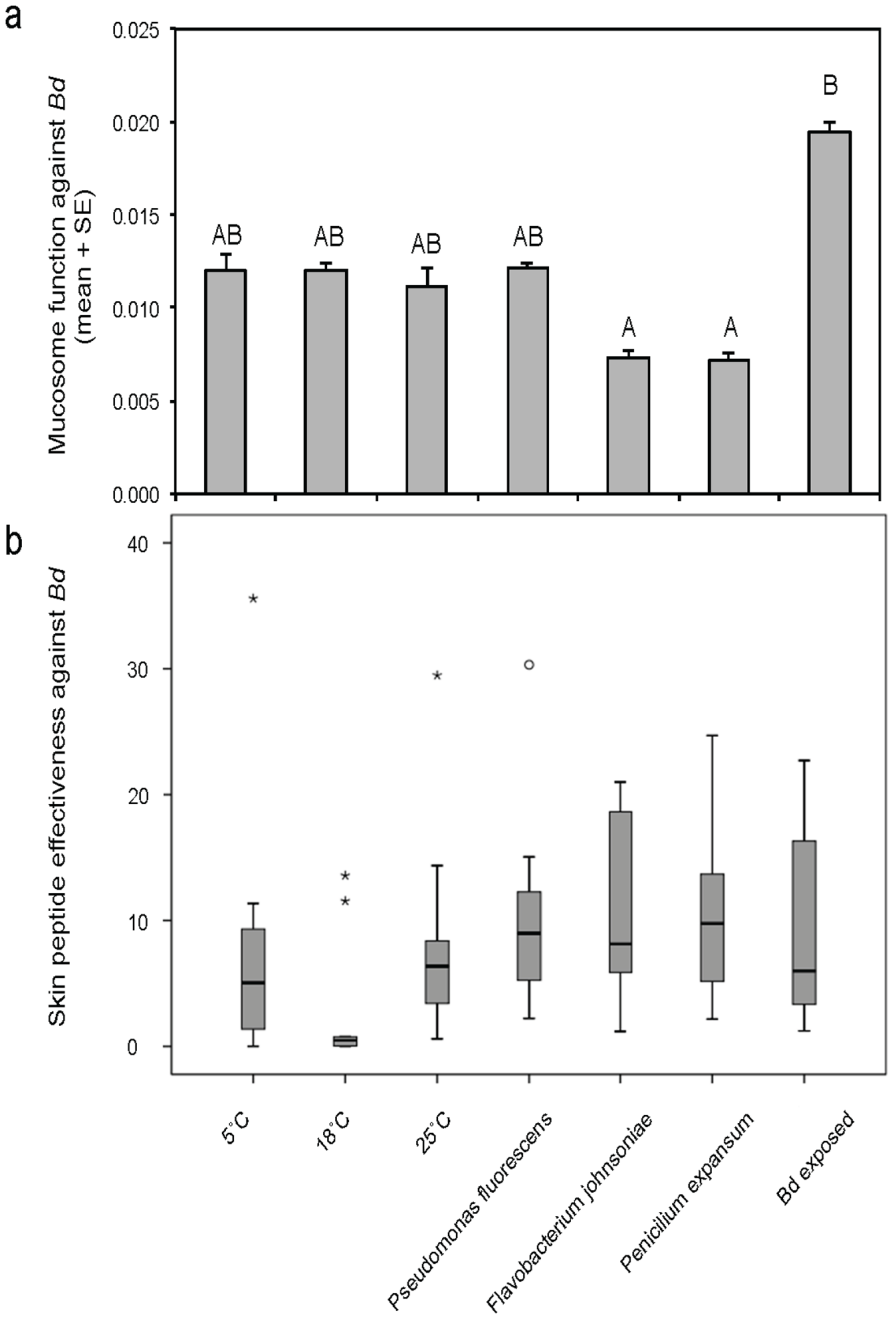
Temperature and probiotic treatments of recently metamorphosed midwife toads, *A. obstetricans*, influence skin mucosome function (a) but not induced skin peptide defenses (b). (a) Mucosome function indicates *B. dendrobatidis* (*Bd*) viability after exposure to amphibian mucus quantified by green: red fluorescence. Significantly different subsets are indicated by letters above bars (Tukey post-hoc test). *Bd* zoospore viability was reduced after exposure to mucus from frogs treated with the bacterium *F. johnsoniae* and the fungus *P. expansum*, and zoospore viability was highest after exposure to mucus from toads previously exposed to *Bd*. (b) Skin peptide effectiveness against *Bd* did not differ significantly among treatments (ANOVA, F^6^ = 0.952, *P* = 0.466).

**Figure 4:**
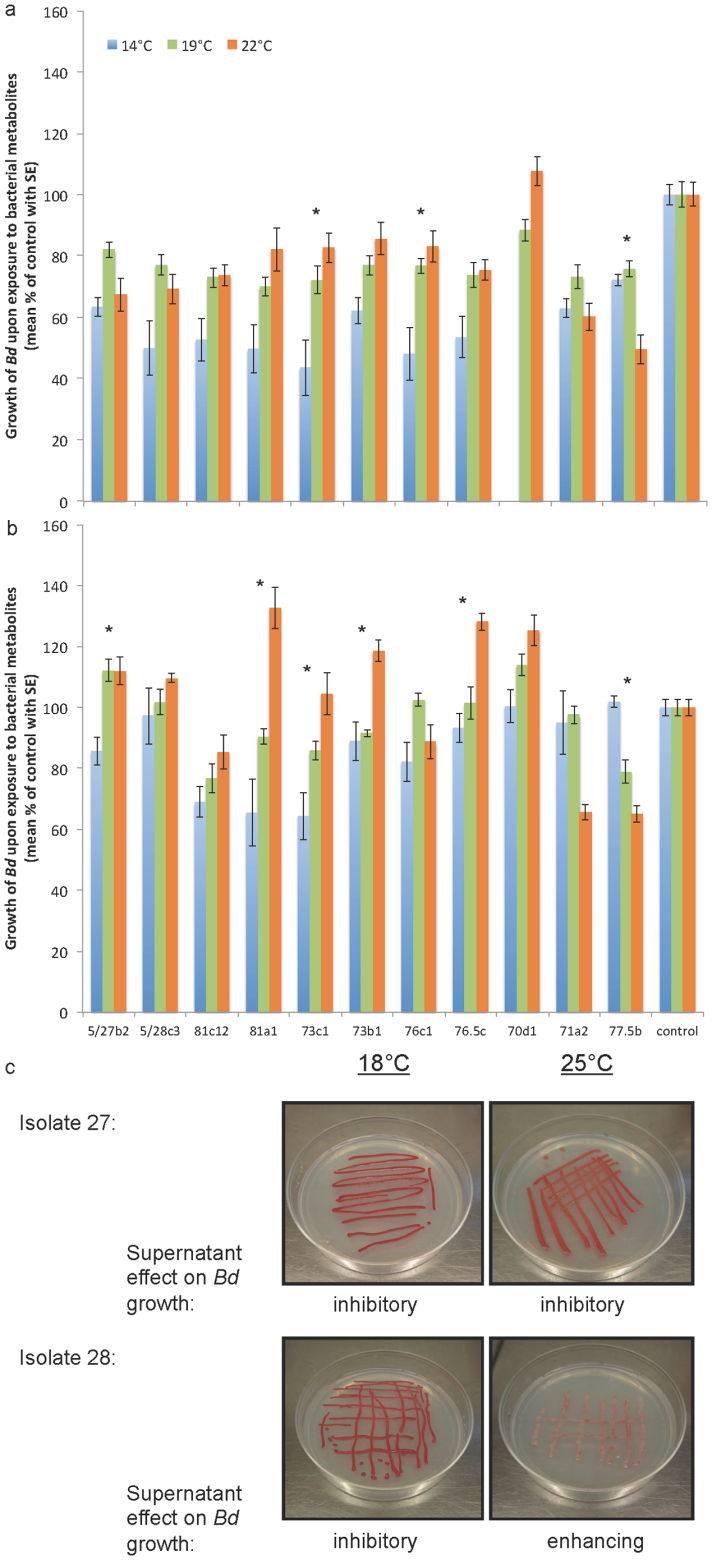
Environmental context determines antifungal capacity of probiotics. Tested temperatures (14, 19, 22°C) significantly affected the production of bacterial metabolites in liquid media that could inhibit *B. dendrobatidis* (*Bd*; GPL isolate VMV 813) zoospore growth in a dose-dependent fashion (a  =  full strength metabolites, b = 1∶10 dilution). * indicates that *Bd* growth differed among metabolite temperature treatments (ANOVA, Bonferroni-corrected P's<0.05). (c) Representative replicates are shown of two isolates of *Serratia plymuthica* isolated from egg clutches of common midwife toads, *Alytes obstetricans*, grown on solid media under different temperature conditions. Filtrate from isolate 27 always inhibited growth of *Bd*, but filtrate from isolate 28 inhibited *Bd* growth at 18°C, and enhanced *Bd* growth at 25°C. Filtrate from sterile media (R2A agar supplemented with 1% tryptone) caused enhanced growth of *Bd*. Note that colony color can be an indication of antifungal metabolites such as prodiginines from red *Serratia spp*. [45], [67], but are produced only under certain growth conditions.

Midwife toads, *A. obstetricans,* were treated with various temperature and probiotic therapies and tested for mucosome function. Host context significantly affected mucosome permissiveness or lethality towards *Bd* (Fig. 3a; ANOVA, F_6_ = 41.606, *P*<0.001). *Bd* viability was similar following incubation with mucosome samples from toads at temperatures ranging from 5–25°C. Mucosome samples from toads previously exposed to *Bd* were least effective at killing *Bd* zoospores, while those from toads treated with probiotics *Flavobacterium johnsoniae* and *Penicillium expansum* were most effective at killing zoospores (Fig. 3a). While *Pseudomonas* in general, and the *P. fluorescens* isolate (76.5c) used in this study were often effective at inhibiting *Bd* in co-culture and produced antifungal metabolites across a range of temperatures ideal for *Bd* growth (Fig. 4a, Table S2 in File S1), there was no significant benefit of this probiotic when applied on hosts in terms of increasing mucosome function and reducing *Bd* viability (Fig. 3a).

Because one significant antimicrobial component of *A. obstetricans* skin mucus is antimicrobial peptides (AMPs) [44], we collected peptide skin secretions, quantified them per surface area of the toads and measured their ability to inhibit *Bd* growth at a standardized concentration of 100 µg/ml. On average, toads produced 0.25 mg peptide per cm^2^ surface area, and at 100 µg/ml these peptides inhibited *Bd* growth by 48.7%. These values did not differ significantly among treatment groups, nor did a combined measure of skin peptide effectiveness against *Bd* (% * mg/cm^2^, Fig. 3b; Kruskal-Wallis tests, *P*'s>0.05). Thus, skin peptides stored in granular glands were not significantly affected by the 2-week temperature and microbe treatments including previous exposure to *Bd*. There was not a significant correlation between peptide effectiveness and mucosome function against *Bd* (Fig. S5 in File S1; Pearson, χ^2^ = −0.102, *P* = 0.827). Zoospore viability after exposure to mucosome samples was significantly higher in the *Bd*-exposure treatment compared to other treatments (Fig. 3a). However, skin peptides induced from hosts in the *Bd*-exposure treatment were effective at inhibiting *Bd* growth, and not significantly different than peptides from toads in other treatments (Fig. 3b).

### Temperature, competition of probiotic strains, and co-culture with *Bd*

Environmental conditions affected the capacity of probiotic bacteria to inhibit the fungal pathogen *Bd* (Table S2 in File S1). Two *Serratia plymuthica* isolates (isolates 27 and 28) were capable of inhibiting *Bd* growth when incubated at 18°C. Isolate 27 was inhibitory under all tested conditions: 18°C, 25°C, and 18°C co-cultured with *Bd*. Isolate 28 significantly enhanced *Bd* growth at 25°C, and was neither enhancing nor inhibitory at 18°C when co-cultured with *Bd* (Fig. 4c, Table S2 in File S1). A dose-response of *Bd* growth inhibition was found such that filtrate diluted 1/10 was significantly less inhibitory than undiluted filtrate (paired t-test, t_35_ = 9.836, *P*<0.001), and filtrate from control plates with or without *Bd* significantly enhanced *Bd* growth (Table S2 in File S1). Testing metabolites of the bacteria growing at 14, 19, and 22°C in liquid culture against the global panzootic lineage of *Bd* showed similar results including a dose-response (Fig. 4a,b, paired t-test, t_31_ = −10.607, *P*<0.001). In several cases, *Bd* growth was enhanced with addition of diluted bacterial metabolites in comparison to positive control growth with RIIA media only (>100%, Fig. 4b). Most cultures were more inhibitory of *Bd* at the lower temperatures, except for *J. lividum*, (isolate 77.5b) which was most inhibitory at 22°C (Fig. 4a,b).

While all bacteria were unique based on 16S rRNA gene sequencing when clustered at 99% similarity, probiotic physiology and function against *Bd* did not always correspond to OTU clustering at 97% similarity (Table S1 in File S1). In other words, bacterial isolates considered to be the same “species” based on 16S rRNA could have different antifungal function. Here, only one of two *Flavobacterium johnsoniae* isolates inhibited *Bd* growth. When grown together, the filtrate remained inhibitory. However, when grown together and co-cultured with *Bd*, the filtrate was no longer inhibitory. Three *Pseudomonas* isolates were capable of inhibiting *Bd* growth, and were inhibitory when combined with or without co-culture with *Bd*. The above mentioned growth inhibition of *Bd* caused by bacterial filtrate was significantly different from control bacterial growth with water only added (independent t-tests, *P*'*s*<0.05 and replicated result; all data shown in Table S2 in File S1). These conditions represent infected or uninfected hosts and are illustrative rather than comprehensive of all possible environmental conditions and competitive interactions.

### Effects of host skin peptides and *Bd* metabolites on probiotics in culture

Amphibian skin defense peptides may regulate the skin microbiota. We found that natural mixtures of skin peptides from *A. obstetricans* at a concentration of 100 µg/ml significantly inhibited growth of *Pseudomonas migulae* (73b1) and significantly enhanced growth of *P. filiscindens* (73c1), *Flavobacterium johnsoniae* (70d1), and *Janthinobacterium lividum* (76.5c; t-test, Bonferroni corrected P's<0.05; Fig. S6 in File S1).

We tested for a direct effect of *Bd* metabolites on bacterial growth, and found that filtrate from two-week old cultures of *Bd* in 0.5% tryptone significantly inhibited the growth of *Serratia plymuthica* (5/27b2, 5/28a3), *F. johnsoniae* (81a1, 70d1), and *P. filiscindens* (73c1), while significantly enhancing the growth of *J. lividum* (77.5b1; t-test, Bonferroni corrected P's<0.05; Fig. S6 in File S1).

## Discussion

We found that a holistic measure of mucosome function against *Bd* is predictive of infection risk in natural populations of amphibians and survival in laboratory exposure experiments. While induced antimicrobial peptides may explain some variation in infection risk (Fig. 1b,d), mucosome function can be altered through probiotic therapy (Fig. 3a), and thus microbial communities play a major role in determining susceptibility to infection with *Bd*. In particular, tadpoles of the endangered midwife toad, *A. obstetricans* may be most at risk of both infection and subsequent disease-induced mortality upon metamorphosis (Fig. 2), even though adult toads are well protected by the mucosome and perhaps resistant to colonization with *Bd*. Similarly, the common frog *R. temporaria* has strong mucosome activity against *Bd*, shows *Bd* colonization resistance, but has relatively poor skin defense peptides. This suggests that this common species has protective microbial communities. Adaptive defenses are not suspected because frogs were raised from eggs and had no history of exposure to *Bd*.

In this study, we provide several striking examples showing that probiotic capacity depends on immunological and environmental context. These examples lead to recommendations for choosing probiotics based on predictable host conditions. Temperature is known to influence amphibian host immune function [41] and bacterial growth, metabolism, pigment and antibiotic production [45]. However, it was surprising that a shift from 18 to 25°C, a typical natural range for midwife toads, caused a common bacterial symbiont of the eggs and skin, *Serratia plymuthica*, to change from inhibiting *Bd* to enhancing *Bd* growth (Fig. 4c). Testing metabolites of the bacteria growing at 14, 19, and 22°C in liquid culture against the global panzootic lineage of *Bd* showed similar results (Fig. 4b). Functional changes in probiotic activity with shifts in temperature have not previously been reported. Our results provide an alternative mechanistic explanation for patterns of susceptibility related to climate, which have previously been limited to empirical observation and pathogen-centered effects [46]–[49].

The microbial interactions we tested also altered antifungal effects relative to what would be predicted from individual isolates. For example, co-culture of *Flavobacterium johnsoniae* with *Bd* caused cultures of the bacterium that normally produce antifungal metabolites to switch off antifungal activity: when grown together with *Bd*, *F. johnsoniae* filtrate was benign, and indeed *Bd* filtrate inhibited the growth of two out of three *F. johnsoniae* isolates (Fig. S6 in File S1). Co-evolution of *Bd* and amphibian hosts is a postulated driver of pathogenicity factors including compounds suppressing host immune defenses [43], [50], [51]. These factors may extend to inhibiting certain antifungal symbionts or altering their function.

Myriad microbial and immune interactions occur once probiotics are added to living hosts. Thus, testing probiotics *in vivo* is critical for testing the intended antifungal effect of probiotic therapy under realistic environmental conditions. We found that previous exposure to *Bd* may have a negative effect on host immunity or the ability of the mucosome to kill zoospores (Fig. 3A). This result is consistent with a study on Australia green-eyed tree frogs, *Litoria serratia*, showing inhibition of ambient skin peptides with *Bd* infection but no inhibition of inducible stored skin peptides [43]. Because stored skin defense peptides can have potent activity against *Bd*, yet not be active on the skin, induced skin peptides may not accurately predict infection susceptibility. This mystery of how seemingly well-defended species can be affected by chytridiomycosis [52] deserves careful study on the conditions under which host skin defense peptides are activated. Induced skin defense peptides were previously used to predict disease susceptibility in Panama [11] and New Zealand [53]. In Panama, most species had weak peptide defenses and declined after disease emergence while only two species had strong peptide defenses against *Bd* compared to reference species of known disease resistance. Of these two species, the one with the highest levels of skin peptide defenses persisted at the field site (*Espadarana prosoblepon*) [54], and the other species (*Agalychnis lemur*) disappeared, but a relict population has been detected nearby (Julie Ray, pers. comm.). In New Zealand, all native species demonstrated high levels of skin peptide defenses and appear to resist chytridiomycosis [53], although populations are in decline [55].

We found that a bacterium *F. johnsoniae* and a fungal probiotic *P. expansum* can increase the *Bd* killing function of the mucosome. The bacterium *P. fluorescens* did not show this effect. Because host AMPs did not appear to be affected by these treatments (Fig. 3B), the observed effects are most likely caused by antifungal metabolites produced by the microbes growing on the amphibian skin [56]. Upregulation of host mucosal immunity excluding AMPs is an untested alternative mechanism, and potentially a beneficial host response to probiotics. A non-responsive immune system when given probiotics may be preferred from a conservation management standpoint in order for the probiotics to colonize the host, establish within the microbiota and persist. However, this in not necessarily common and immune stimulation in response to probiotics occurs in other systems [57], [58].

An ideal probiotic would produce metabolites that inhibit *Bd* growth as shown above, and also be uninhibited by host skin defense peptides. A literature review demonstrates that skin peptides can inhibit the growth of some bacteria, but not others, and suggests that skin defense peptides may be critical in structuring the symbiont community on amphibian skin [52]. Rollins-Smith *et al.* [35] showed that *Aeromonas hydrophila*, a common resident on amphibian skin and also an opportunistic pathogen, could tolerate high levels of host antimicrobial peptides. This organism shows antifungal characteristics including activity against *Bd* growth [33]. The ability of extracellular products of *A. hydrophila* to inhibit amphibian antimicrobial peptides indicates a co-evolutionary relationship between host and symbionts [59]. In addition, *Pseudomonas mirabilis* and *Serratia liquefaciens* were found to be resistant to antimicrobial peptides from several host frog species [60]. Here we used probiotics that largely resisted low concentrations of natural mixtures of host defense peptides (Fig. S6 in File S1). Thus, to increase the likelihood of probiotic establishment, use of probiotics with a co-evolutionary relationship with the target host may be advantageous.

While easily cultured, the isolates tested here may not be dominant community members based on culture-independent analyses [31], [61], [62]. Therefore, future studies will benefit by examining the effects of probiotic treatments on the natural microbial communities on host amphibians using culture-independent techniques such as next-generation sequencing. While community interactions are difficult to test in vitro and before probiotics are applied to a host, our results affirm that testing probiotics under certain foreseeable contexts may increase the pace of biotherapy development.

**Figure 5:**
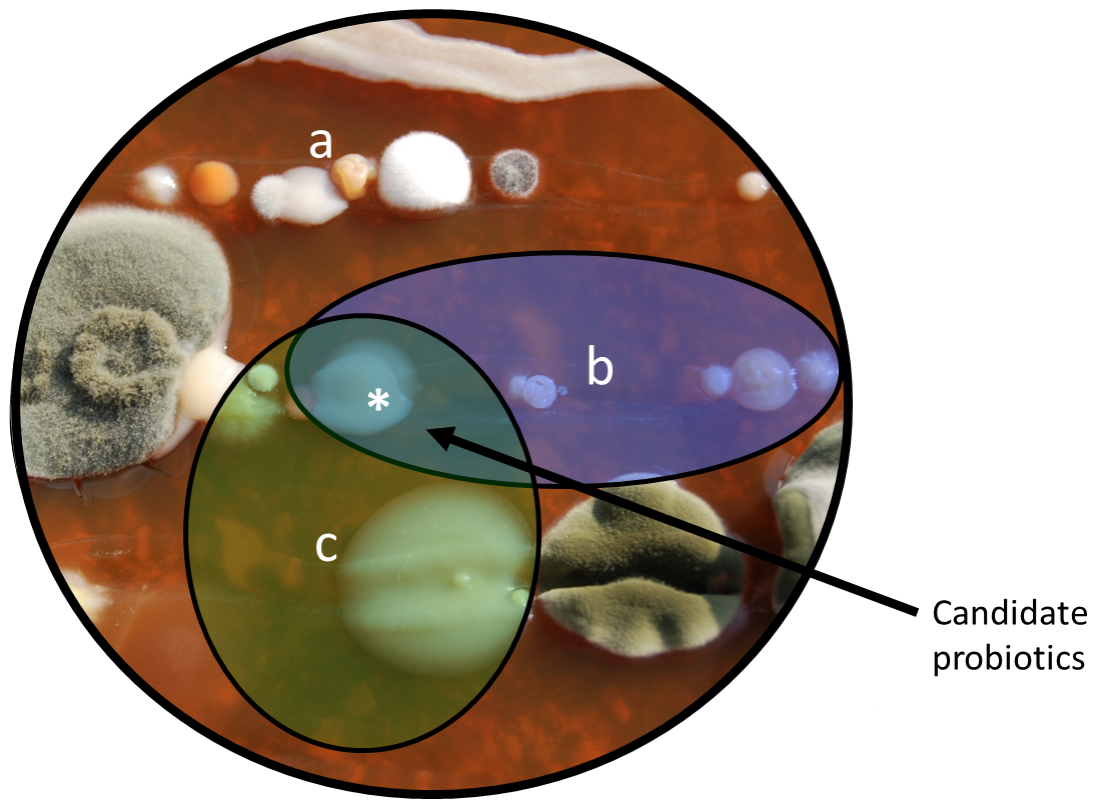
Choosing probiotics with the greatest potential against amphibian chytridiomycosis. Candidate probiotic bacteria (or fungi) are isolated from populations of amphibians that are able to persist in the presence of *B. dendrobatidis* (*Bd*) [1]. To increase the chances of successful prophylactic biotherapy, candidate probiotics should be tested for at least three characteristics: (a) capacity to inhibit *Bd* growth as a pure isolate without specific competitive interactions to induce antifungal metabolites, (b) capacity to inhibit *Bd* at a temperature range consistent with host habitat, and (c) resistance to host skin immune defenses that would complicate probiotic establishment. Remedial biotherapy of already infected individuals should maintain antifungal capacity when grown in competition with *Bd* and withstand the sometimes lethal effects of *Bd* metabolites (Fig. S6 in File S1). Testing probiotic effect in vivo can be accomplished without resorting to pathogen exposure experiments by using the mucosome function assay described here.

Because potential probiotics that inhibit the growth of *Bd* only do so under certain conditions, we recommend the following screening criteria (Fig. 5): (1) Candidates for probiotic development should be chosen from among the culturable microbiota locally present on tolerant hosts or populations that are able to persist with *Bd* [32], [33]. (2) Candidates should have the capacity to inhibit *Bd* growth when grown in isolation, in co-culture with *Bd*, and in an environmental context relevant to the amphibian life-cycle, and (3) the ability to resist immune defenses on host skin, establish within the microbiota, and contribute to antifungal defenses *in vivo*. Resistance to mucosal immune defenses may be critical for establishment within the microbial community associated with the skin, and critical for long-term persistence. Some symbionts appear to be assisted in surviving on the host by thriving on skin mucosal products. Mucosal oligosaccharides, for example, differ among hosts and life-history stages, and may be a selective force in structuring the microbiota [63], [64]. Amphibian skin provides a useful model of host-microbiota interactions to better understand mechanisms of microbial community assembly and maintenance within vertebrate mucosa. Indeed, these mechanisms underlie strategies to promote human health by manipulating microbial communities - a long-term goal of the Human Microbiome Project [7], [65].

While screening for candidate probiotics, some beneficial organisms may be inadvertently discarded based on tests of bacterial filtrate on *Bd* growth. Microbes producing antifungal metabolites such as bacteriocins [66] or small molecule antibiotics [56], [67] will be detected by this method. However, microbes may also compete directly for space or resources, and may exclude pathogenic fungi by other mechanisms [26], [68]. Furthermore, microbial secondary metabolites such as prodiginines produced by *Serratia spp*. can be immunosuppressive [67]. Probiotics may strongly influence host immunity through interactions with host Toll-like receptors or NOD-like receptors, or through interactions with epithelial cells and immune system cells modulating both local and systemic immune responses [69]. The immunomodulatory effect of probiotics cannot be tested with *in vitro Bd* growth assays and host trials are necessary to test for these emergent properties of probiotics.

Antimicrobial peptides and a range of other defenses protect amphibian skin by synergizing or interacting with microbes [41], [70]. Thus, a better indication of antifungal effect of probiotics was obtained by testing the mucosome directly on zoospore viability. *In vitro* screening cannot incorporate every factor and eventually *in vivo* trials, both in the lab and under natural conditions are necessary to determine if an overall health benefit is provided. However, beginning with a probiotic that is not likely to become an opportunistic pathogen with changing climatic conditions may be a consideration. Transmissible probiotics would aid disease control at the population level [33], and if able to persist through metamorphosis when applied to tadpoles, disease presentation at this critical developmental stage could be avoided for *A. obstetricans* and other susceptible amphibians [40]. Additionally, *Bd* metabolites are known to be toxic to amphibian lymphocytes [50], and in this study were toxic to certain bacteria such as *Serratia plymuthica* (Fig. S6 in File S1), perhaps prohibiting the use of certain probiotics intended as remedial biotherapy for infected individuals. The potential for negative biodiversity-function relationships, especially among mixtures of closely related bacteria, cautions against the use of probiotic mixtures that may cause interference competition and reduce host protection [71]. Further refinements to the probiotic screening and discovery process will incorporate next-generation sequencing analyses to target rare or as yet uncultured microbes of interest, and testing microbial consortia that appear linked to disease resistance function. Measuring the effectiveness of applied probiotics is a second step in managing disease risk.

No previous studies have attempted to relate skin microbiota or a holistic measure of skin defense function against *Bd* with disease susceptibility. Given the extreme complexity of the skin micro-ecosystem and interactions described above, the holistic measure of mucosome function presents a significant advance in our capacity to predict relative disease susceptibility, and to measure the success of managed treatments without resorting to infection trials. Here, we examined overall prevalence of infection in Switzerland and Europe and test for correlations at these broad scales with innate defenses from selected life-stages and species (Fig. 1). We found a very strong correlation between mucosome function against *Bd* and infection prevalence in the field and upon experimental exposure. Since Bd-naïve amphibians were sampled for mucosome function, adaptive immunity such as mucosal antibodies is not indicated and antifungal function can be attributed primarily to innate defenses including the microbiota. Indeed, altering the microbiota through probiotic treatments affected mucosome function against *Bd*. In addition to assessing infection risk in natural amphibian assemblages, mucosome functional assays can now be used to assess risk in relict populations or in captive colonies slated for reintroduction. While the efficacies of human probiotics are under scrutiny [2], quantifying the effectiveness of amphibian probiotic treatments under scenarios of changing environmental conditions is a tangible goal.

## Supporting Information

File S1 Protocol for determining *Bd* viability, supplementary tables and figures.
(PDF)
