# Correction: Interacting Symbionts and Immunity in the Amphibian Skin Mucosome Predict Disease Risk and Probiotic Effectiveness

**DOI:** 10.1371/journal.pone.0104590

**Published:** 2014-07-30

**Authors:** The PLOS ONE Staff

There is a missing affiliation for co-author, Jos Kielgast. The correct affiliations are:

Section for Freshwater Biology, Department of Biology, University of Copenhagen, Copenhagen, Denmark

Center for Macroecology, Evolution and Climate Natural History Museum of Denmark Universitetsparken 15, DK-2100, Denmark

## References

[1] Woodhams DC, Brandt H, Baumgartner S, Kielgast J, Küpfer E, et al (2014) Interacting Symbionts and Immunity in the Amphibian Skin Mucosome Predict Disease Risk and Probiotic Effectiveness. PLoS ONE 9(4): e96375 doi:10.1371/journal.pone.0096375 24789229 10.1371/journal.pone.0096375 PMC4005770

