# Supplementary material for: Interacting Symbionts and Immunity in the Amphibian Skin Mucosome Predict Disease Risk and Probiotic Effectiveness
Source: PLoS One. 2014 Apr 30;9(4):e96375. doi: 10.1371/journal.pone.0096375 (PMC4005770; doi:10.1371/journal.pone.0096375)
Supplement: File S1 — Protocol for determining Bd viability, supplementary tables and figures. (PDF) [file pone.0096375.s001.pdf]

Supporting Information

Interacting symbionts and immunity in the amphibian skin mucosome predict disease risk and probiotic effectiveness

Contents:

I. Protocol for determining *Bd* viability
II. Supplementary Tables
Table S1. Bacterial isolates and accession numbers
Table S2. Data table for environmental context experiments
III. Supplementary Figures
Figure S1. Standard curve for determining *Bd* viability
Figures S2, S3. Kaplan-Meier survivorship curves
Figures S4, S5. Correlations of mucosome function and skin peptide effectiveness against *Bd*
Figure S6. Growth of bacteria with skin peptides or *Bd* filtrate
IV. Supplementary References

I. Protocol for determining *Bd* viability

Plate preparation –

Wash *Batrachochytrium dendrobatidis* (*Bd*) zoospores from agar plates (4-5 d old cultures) with 3 ml Tris-buffered saline (TBS, pH 7.4) per plate 15-20 min. Collect zoospores in tube and determine density by haemocytometer count using Lugol solution (Sigma) to kill and stain zoospores (90 µl Lugol solution, 10 µl zoospores). Dilute to 10^6 zoospores ml^{-1}. Add 50 µl zoospores to each well of 96-well flat-bottom plate, except for negative control wells. For negative control wells, heat-kill zoospores at 60˚C for 10 min or flash freeze in liquid nitrogen. We suggest replicates of 4 wells per sample and 8 wells per control or standard. A standard curve can be generated by adding various proportions of live:dead zoospores (Supplementary Fig. S1). Cover plates to minimize evaporation. Sterile conditions are not necessary.

Wash preparation –

Weigh amphibians, calculate surface area according to Spight (1968; salamanders) or McClanahan & Baldwin (1969; frogs).

To convert frog mass to surface area: surface area in cm^2 =9.9*(mass in g)^(0.56)

To convert salamander mass to surface area: surface area in cm^2 =8.42*(mass in g)^(0.694)

Divide surface area by 4 to determine quantity of water to add to the amphibian for the wash (0.25 ml per 1 cm^2 surface area). Rinse amphibian for 1 hr. Remove amphibian, place wash solution on ice. Add 50 µl wash solution per well in 96-well plate (4 wells per amphibian wash sample).

Alternatively, a syringe filter can be used to filter skin wash or water from the amphibian habitat to remove live bacterial cells. The filtrate can than be tested as above, or in a standard fungal growth assays (Rollins-Smith et al., 2002).

Stain preparation-

Use the LIVE/DEAD Sperm viability kit (Molecular Probes, L-7011). Dilute stock SYBR 14 dye (1mM SYBR 14 in DMSO) 1:100 in 1X TBS.

1 plate = 96 wells x 6 µl = 576 µl diluted SYBR 14 needed.

580/100 = 5.8 µl stock + 574.2 µl TBS

Use Propidium iodide undiluted.

Assay –

Add 50 µl water to control wells and standard wells. Add 50 µl wash solution to sample wells. You should have 100 µl total volume in all wells. Incubate 1 hr. Add 6 µl diluted SYBR 14 per well. Cover from light and incubate 10 min. Add 4 µl undiluted Propidium iodide, cover, and incubate 10 min.

Read plates according to kit instructions. For green fluorescence (SYBR 14) this is approximately (excitation-emission) 485-528 nm, and for red fluorescence (Propidium iodide) this is 530-645 nm. Calculate proportion of green:red fluorescence. Produce a curve based on the standards, fit a line and use the equation to calculate proportion living zoospores from your proportion fluorescence sample values.

This assay was first adapted for detecting *Bd* viability by Stockwell et al. (2010). Care should be taken when interpreting results of *Bd* viability assays using this method after inducing secretions of large quantities of skin peptides as these concentrations interfere with fluorescence measurements. *Bd* growth assays (see main text) may be a better method to test the effects of induced or concentrated peptides on *Bd*.

An alternative and inexpensive method is to examine *Bd* zoospore viability with a compound microscope after staining with trypan blue dye according to McMahon and Rohr (2014).

II. Supplementary Tables

Table S1. Accession numbers for partial 16S rRNA gene sequences in the European Nucleotide Archive (http://www.ebi.ac.uk/ena/data/view/HE802986-HE802992). All sequences are unique at 99% similarity as determined by the UCLUST algorithm in QIIME (Caporaso *et al.*, 2010).

Accession number	OTU (97% similarity)	Organism	Isolate name	Isolation source
HE802986	1	*Serratia plymuthica*	5/27b2	eggs of *Alytes obstetricans*
HE802987	1	*Serratia plymuthica*	5/28a3	eggs of *Alytes obstetricans*
HE802988	2	*Flavobacterium johnsoniae*	81c12	adult female *Alytes obstetrican*
HE802989	3	*Flavobacterium johnsoniae*	81a1	adult female *Alytes obstetrican*
HG313637	3	*Flavobacterium johnsoniae*	70d1	adult male *Alytes obstetricans*
HE802990	4	*Pseudomonas filiscindens*	73c1	adult male *Alytes obstetricans*
HE802992	4	*Pseudomonas plecoglossicida*	76c1	adult male *Alytes obstetricans*
HE802991	5	*Pseudomonas migulae*	73b1	adult male *Alytes obstetricans*
HG313686	6	*Pseudomonas fluorescens*	76.5c	eggs of *Alytes obstetricans*
HG752431	6	*Pseudomonas sp.*	71a2	adult *Alytes obstetricans*
HG313693	7	*Janthinobacterium lividum*	77.5b1	eggs of *Alytes obstetricans*

Table S2. Environmental context experiments including bacterial isolates grown under various competitive and temperature conditions. Filtered metabolites washed from experimental R2A with 1% tryptone agar plates were added to *Batrachochytrium dendrobatidis* (*Bd*; Swiss isolate TG 729) zoospores in 96-well plates. *Bd* was monitored for growth and significant growth inhibition or enhancement is reported as well as non-significant changes (ns, Independent t-test, $P > 0.05$).

Experiment	Treatment	*Bd*	Temperature (˚C)	Metabolite effect on *Bd*
1	Control		18	enhancing
2	Control		18	enhancing
3	Control	+	18	ns
4	Control	+	18	enhancing
5	*Serratia plymuthica* isolate 27		18	inhibitory
6	*Serratia plymuthica* isolate 27		18	inhibitory
7	Serratia plymuthica isolate 28		18	inhibitory
8	*Serratia plymuthica* isolate 28		18	inhibitory
9	*Serratia plymuthica* isolate 27		25	ns
10	*Serratia plymuthica* isolate 27		25	inhibitory
11	*Serratia plymuthica* isolate 28		25	enhancing
12	*Serratia plymuthica* isolate 28		25	enhancing
13	*Serratia plymuthica* isolate 27	+	18	ns
14	*Serratia plymuthica* isolate 27	+	18	inhibitory
15	*Serratia plymuthica* isolate 28	+	18	ns
16	*Serratia plymuthica* isolate 28	+	18	ns
17	*Flavobacterium johnsoniae* 81c12		18	inhibitory
18	*Flavobacterium johnsoniae* 81c12		18	inhibitory
21	*Flavobacterium johnsoniae* 81a1		18	ns
22	*Flavobacterium johnsoniae* 81a1		18	ns
23	*Flavobacterium johnsoniae* both isolates		18	inhibitory
24	*Flavobacterium johnsoniae* both isolates		18	inhibitory
25	*Flavobacterium johnsoniae* both isolates	+	18	ns
26	*Flavobacterium johnsoniae* both isolates	+	18	ns
27	*Pseudomonas filiscindens* 73c1		18	ns
28	*Pseudomonas filiscindens* 73c1		18	inhibitory
29	*Pseudomonas migulae* 73b1		18	inhibitory
30	*Pseudomonas migulae* 73b1		18	inhibitory
31	*Pseudomonas plecoglossicida* 76c1		18	inhibitory
32	*Pseudomonas plecoglossicida* 76c1		18	ns
33	*Pseudomonas* all three isolates		18	ns
34	*Pseudomonas* all three isolates		18	inhibitory
35	*Pseudomonas* all three isolates	+	18	inhibitory
36	*Pseudomonas* all three isolates	+	18	inhibitory
37	*Pseudomonas fluorescens* 76.5c		18	ns
38	*Pseudomonas fluorescens* 76.5c		18	ns
39	*Pseudomonas fluorescens* 76.5c		18	inhibitory

III. Supplementary Figures

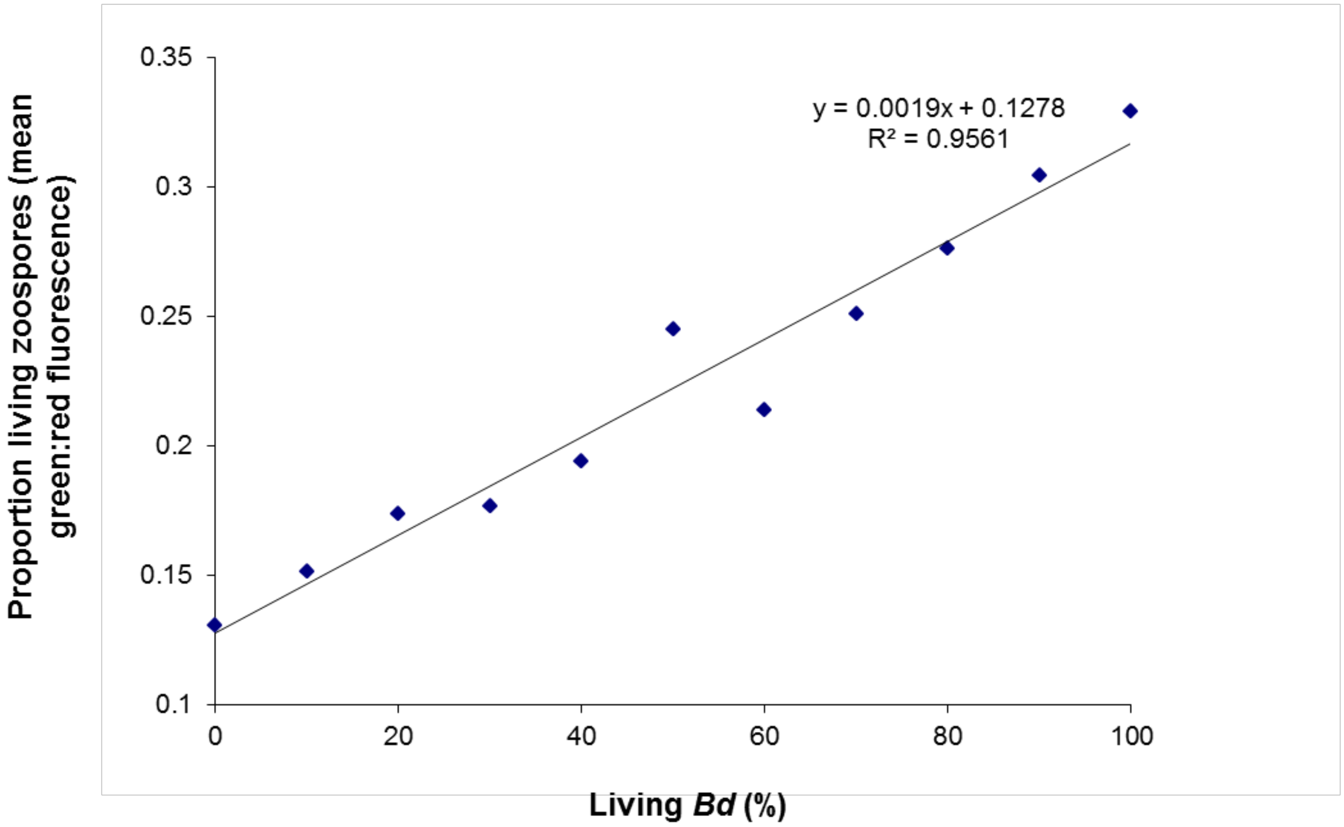

Figure S1. Standard curve showing ratio of fluorescence at various concentrations of living zoospores produced by combining live zoospores and heat-killed zoospores in Tris-buffered saline.

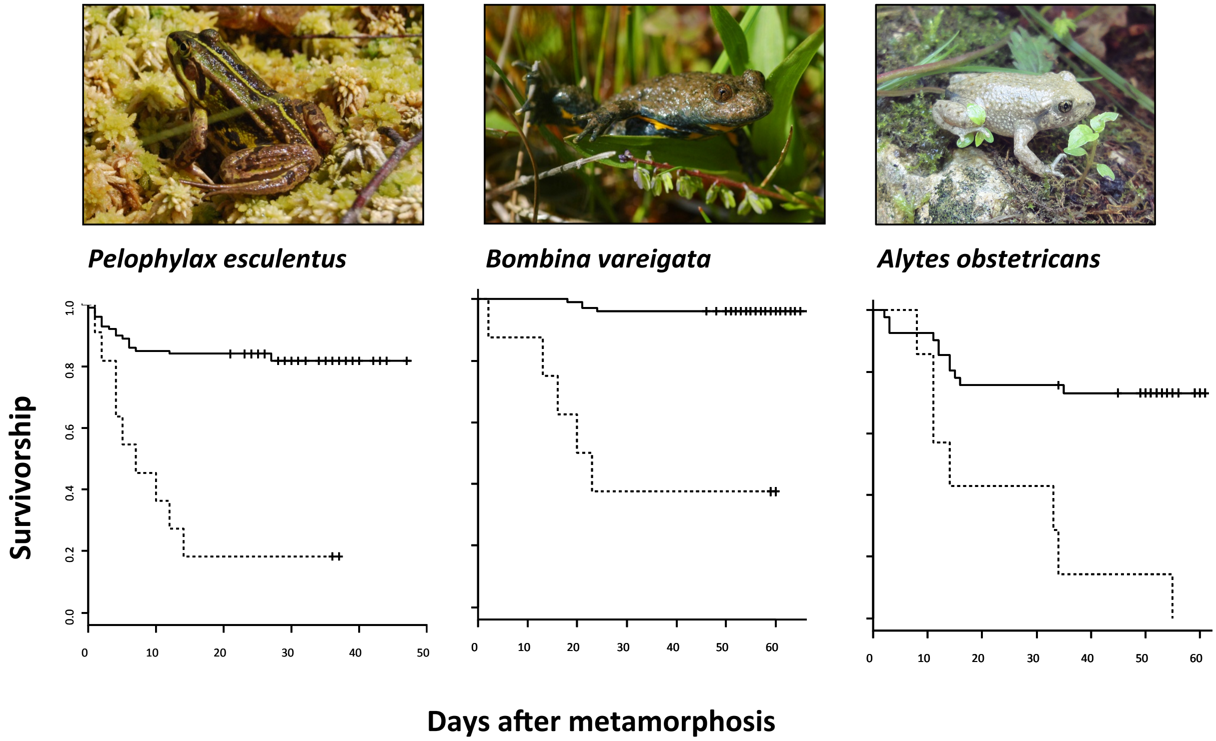

Figure S2. Kaplan-Meier survivorship curves for uninfected (solid lines) and infected (dashed lines) tadpoles upon metamorphosis. Small vertical lines indicate individuals that survived until removal from the experiment. Inoculation dose was not found to influence infection or survival outcomes (Baumgartner, 2009), thus dose treatments were combined for analysis.

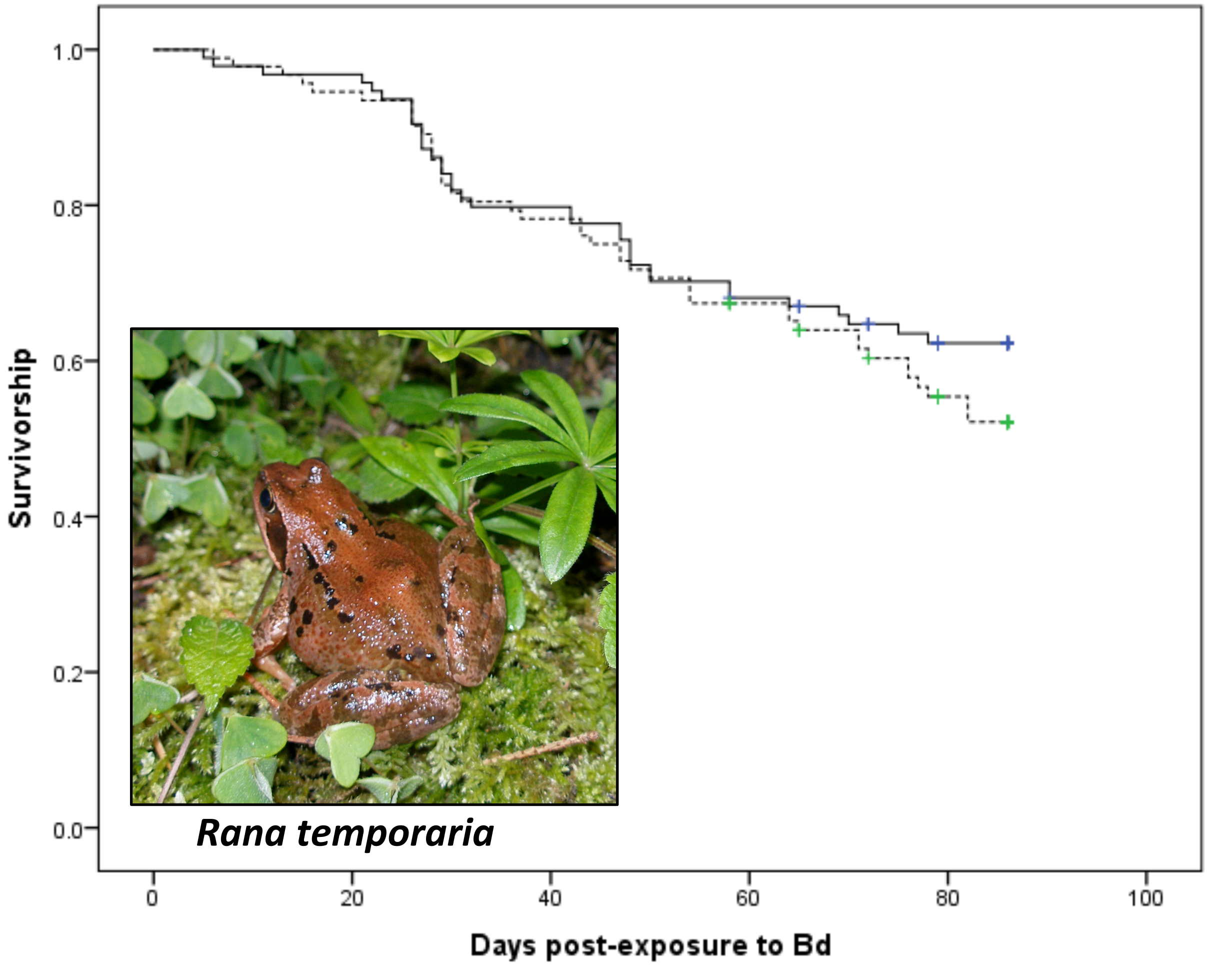

Figure S3. Kaplan-Meier survivorship curves for control (solid lines) and exposed (dashed lines) *Rana temporaria* exposed to *Bd* at metamorphosis. Small vertical lines indicate individuals that survived until removal from the experiment. Additional experimental details are found in Küpfer (2010).

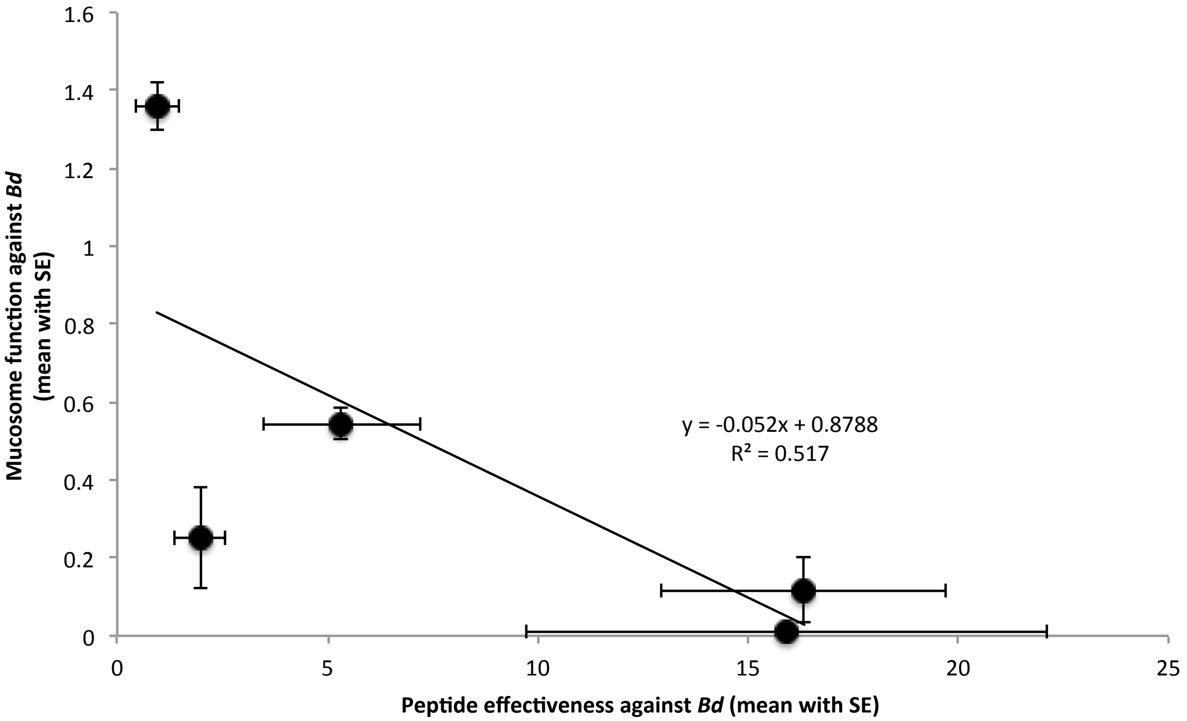

Figure S4. Correlation of skin peptide effectiveness against *Bd* and viability of *Bd* after exposure to amphibian mucus (mucosome function) including adults of five amphibian species from Switzerland indicating that skin peptides explain some of the variation in mucosome function.

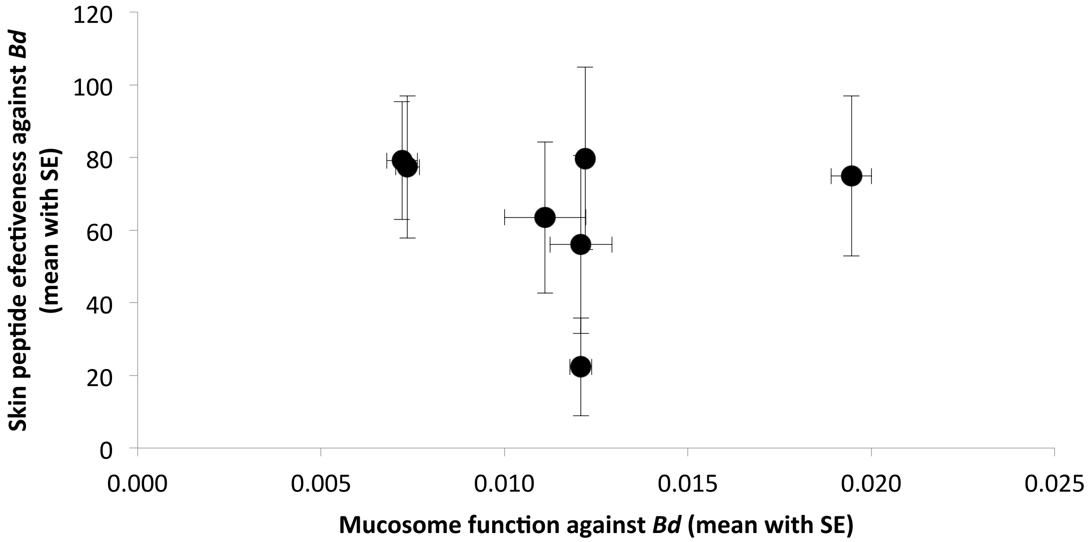

Figure S5. Correlation of skin peptide effectiveness against *Bd* and viability of *Bd* after exposure to amphibian mucus (mucosome function) including *Alytes obstetricans* from seven experimental treatments. Skin peptide effectiveness did not differ significantly among treatments and the far right outlier indicates frogs exposed to *Bd* with suppressed mucosome function. When the *Bd*-exposed frogs are excluded, *Bd* viability after mucosome exposure shows a negative correlation with skin peptide effectiveness.

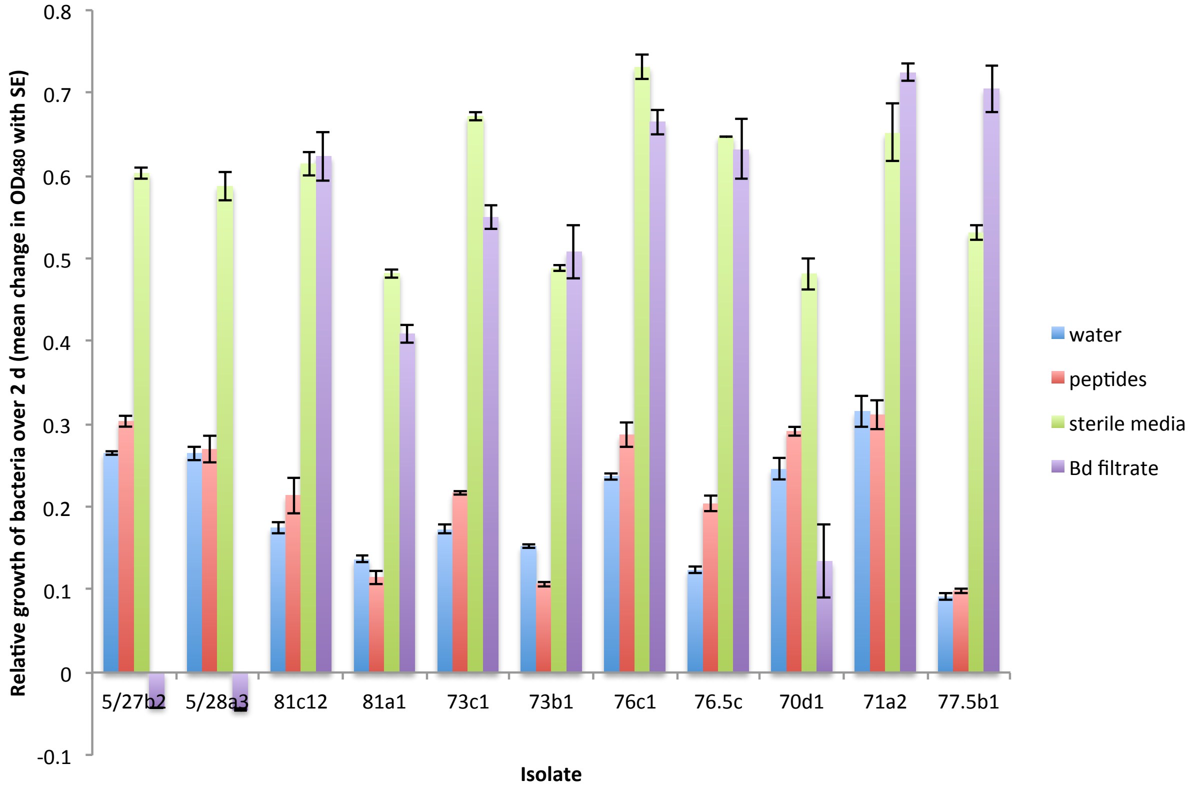

Figure S6. Growth of bacteria over 2 d measured by change in optical density at 480nm (OD_{480}). Water is the control for peptides, and sterile media (0.5% tryptone) is the control for *Bd* filtrate from GPL type isolate JEL197. Skin peptides from *A. obstetricans* (100 µg/ml) significantly inhibited growth of *Pseudomonas migulae* (73b1) and significantly enhanced growth of *P. filiscindens* (73c1), *Flavobacterium johnsoniae* (70d1), and *Janthinobacterium lividum* (76.5c; t-test, Bonferroni corrected P's < 0.05). Filtrate from two-week old cultures of *Bd* significantly inhibited the growth of *Serratia plymuthica* (5/27b2, 5/28a3), *F. johnsoniae* (81a1, 70d1), *P. filiscindens* (73c1), while significantly enhancing the growth of *J. lividum* (77.5b1).

IV. Supplementary References

Baumgartner S (2009) Experimental infection of three native European amphibian larvae by the pathogen *Batrachochytrium dendrobatidis*. Master's thesis, University of Zurich.

Caporaso JG, Kuczynski J, Stombaugh J, Bittinger K, Bushman FD, et al. (2010) QIIME allows analysis of high-throughput community sequencing data. Nat Meth 7: 335-336.

Daum JM, Davis LR, Bigler L, Woodhams DC (2012) Hybrid advantage in skin peptide immune defenses of water frogs (*Pelophylax esculentus*) at risk from emerging pathogens. Infect Genet Evol 12: 1854-1864.

Küpfer E (2011) Anthropogenic stress reduces fitness and antimicrobial immune defence in common frogs (*Rana temporaria*). Master's thesis, University of Zurich.

McClanahan LJ, Baldwin R (1969) Rate of water uptake through the integument of the desert toad, *Bufo punctatus*. Comp Biochem Physiol 28: 381–389.

McMahon TA, Rohr JR (2014). Trypan blue dye is an effective and inexpensive way to determine the viability of *Batrachochytrium dendrobatidis* zoospores. Ecohealth DOI: 10.1007/s10393-014-0908-0.

Rollins-Smith LA, Doersam JK, Longcore JE, Taylor SK, Shamblin JC, et al. (2002) Antimicrobial peptide defenses against pathogens associated with global amphibian declines. Dev Comp Immunol 26: 63-72.

Spight TM (1968) The water economy of salamanders: evaporative water loss. Chicago J 41: 195-203.

Stockwell MP, Clulow J, Mahony MJ (2010). Efficacy of SYBR 14/propidium iodide viability stain for the amphibian chytrid fungus *Batrachochytrium dendrobatidis*. Dis Aquat Org 88: 177-181.
